# Exposure of Wild Mammals to Influenza A(H5N1) Virus, Alaska, USA, 2020–2023

**DOI:** 10.3201/eid3104.241002

**Published:** 2025-04

**Authors:** Andrew M. Ramey, Kimberlee B. Beckmen, David T. Saalfeld, Kerry L. Nicholson, Buck A. Mangipane, Laura C. Scott, David E. Stallknecht, Rebecca L. Poulson

**Affiliations:** US Geological Survey Alaska Science Center, Anchorage, Alaska, USA (A.M. Ramey, L.C. Scott); Alaska Department of Fish and Game, Fairbanks, Alaska, USA (K.B. Beckmen, K.L. Nicholson); Alaska Department of Fish and Game, Anchorage (D.T. Saalfeld); National Park Service, Lake Clark National Park and Preserve, Port Alsworth, Alaska, USA (B.A. Mangipane); Southeastern Cooperative Wildlife Disease Study, University of Georgia, Athens, Georgia, USA (D.E. Stallknecht, R.L. Poulson).

**Keywords:** influenza, viruses, vector-borne infections, zoonoses, avian influenza, bird flu, highly pathogenic, serology, Alaska, United States

## Abstract

Serum samples from wild mammals inhabiting Alaska, USA, showed that 4 species, including *Ursus arctos* bears and *Vulpes vulpes* foxes, were exposed to influenza A(H5N1) viruses. Results indicated some mammals in Alaska survived H5N1 virus infection. Surveillance efforts may be improved by incorporating information on susceptibility and detectable immune responses among wild mammals.

The panzootic of goose/Guangdong lineage highly pathogenic avian influenza (HPAI) A(H5N1) clade 2.3.4.4b has resulted in unprecedented impact to animal health. Numerous reports have described the geographic scope of disease, identified affected species, and reconstructed spatiotemporal dissemination patterns (*1*–*7*). Infection patterns remain cryptic, particularly among wildlife. For example, little or no quantitative information on the number and species composition of wild animals susceptible to and infected with HPAI H5N1 clade 2.3.4.4b viruses is available for most global regions. Even less information is available regarding prior exposure of wildlife and recovery from infection. Such information is critical for clarifying the evolutionary pressures, epidemiologic patterns, and risks associated with those viruses. We aimed to fill data gaps pertaining to the exposure of wildlife to HPAI H5N1 clade 2.3.4.4b viruses by using serum samples opportunistically collected from diverse wild mammals inhabiting Alaska, USA.

## The Study

**Figure 1:**
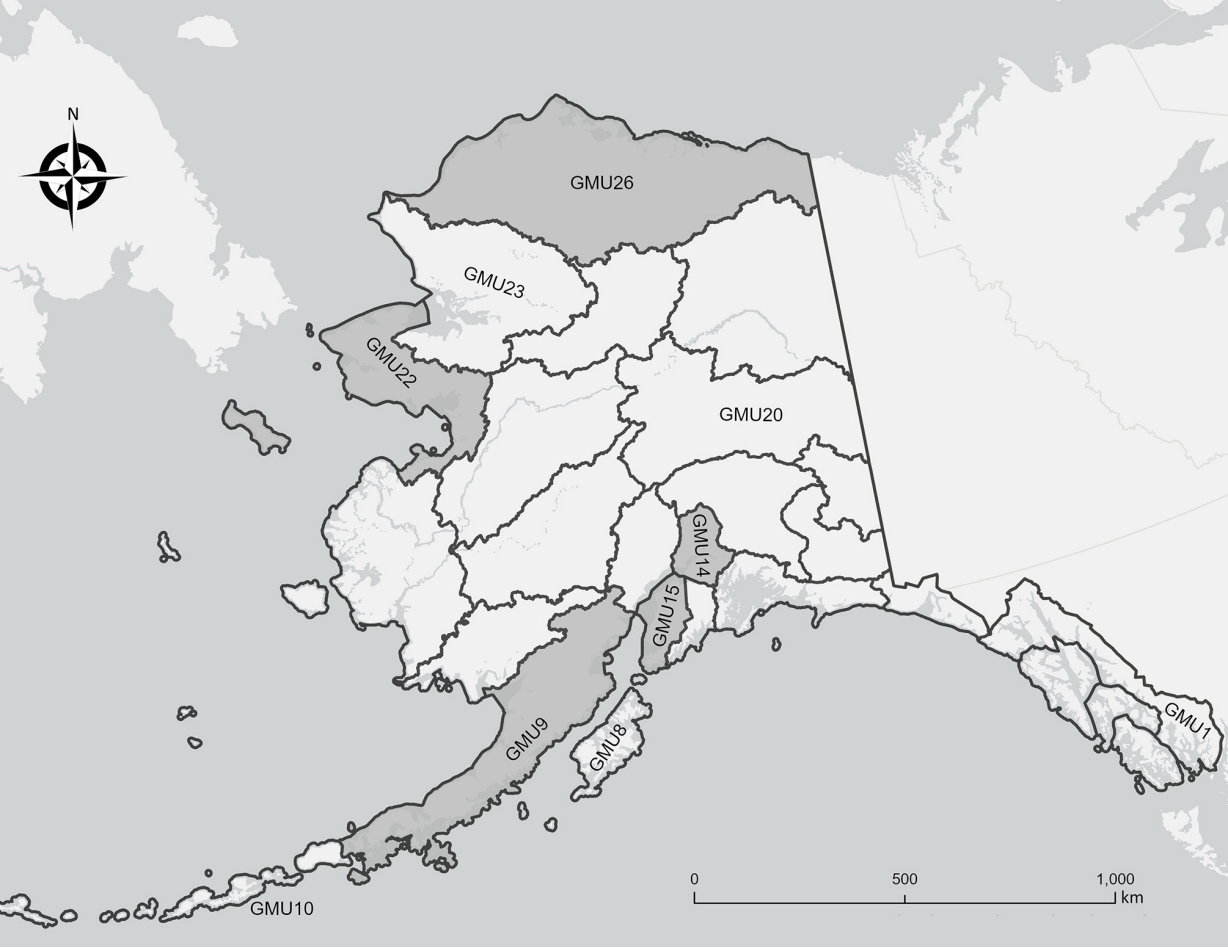
Originating GMUs for 169 serum samples from wild mammals tested for reactivity to influenza A(H5N1) virus, Alaska, USA, 2020–2023. No serum samples were collected from unlabeled GMUs. Gray shading indicates GMUs from which H5 and N1 seropositive samples originated. Locations within GMUs referenced in the text include the Arctic North Slope of Alaska (GMU26), Lake Clark National Park and Preserve (GMU9), and the Northwest Arctic (GMU22). GMU, game management unit.

As part of previously planned biological operations during January 3, 2020–September 2, 2023, we collected 169 serum samples from American mink (*Neovison vison*; n = 2), bearded seals (*Erignathus barbatus*; n = 11), black bears (*Ursus americanus*; n = 9), brown bears (*Ursus arctos*; n = 45), Canada lynx (*Lynx canadensis*; n = 21), coyotes (*Canis latrans*; n = 1), red foxes (*Vulpes vulpes*; n = 41), spotted seals (*Phoca largha*; n = 1), wolves (*Canis lupus*; n = 33), and wolverines (*Gulo gulo*; n = 5) in Alaska (Figure 1). Several agencies collaborated on wildlife surveillance activities, including the Alaska Department of Fish and Game (Institutional Animal Care and Use Committee [IACUC] approval nos. 0062-2019-28, 0005-2020-0028, 0095-2020-0037, 0021-2023-0032, and 0109-2023-0036; https://olaw.nih.gov/resources/tutorial/iacuc.htm), National Oceanic and Atmospheric Administration (National Marine Fisheries Service research permit no. 26254 and IACUC approval no. 0027-2023-0025), and National Park Service (Alaska Department of Fish and Game scientific permit nos. 22-042 and 23-022; IACUC approval no. AK_LACL_Mangipane_Bears_2021.A).

We tested the serum samples for antibodies to the influenza A virus nucleoprotein by using a commercially available blocking ELISA (bELISA), AI MultiS-Screen Ab test, (IDEXX Laboratories, https://www.idexx.com) at the US Geological Survey Alaska Science Center (ASC; Anchorage, Alaska, USA) and the University of Georgia Southeastern Cooperative Wildlife Disease Study (SCWDS; Athens, Georgia, USA). We determined positivity by using the manufacturer’s recommendation of serum/negative (S/N) optical density ratio for poultry (<0.5) and the threshold evidenced to be both sensitive and specific for wild birds (S/N <0.7) (*8*,*9*). We tested all samples with sufficient serum remaining by using hemagglutination inhibition (HI) and virus microneutralization (VN) for antibodies to HPAI H5 clade 2.3.4.4b virus and North American lineage low pathogenicity avian influenza (LPAI) H5 viruses at SCWDS by using previously described procedures (*10*,*11*). We also tested serum samples for reactivity to N1 subtype influenza A viruses by using an enzyme-linked lectin assay (ELLA) at SCWDS, as previously reported (*11*). We determined seropositivity on the basis of HI (>8), VN (>20), and ELLA (>80) titers.

Results for bELISA testing were comparable using the S/N ratios of <0.5 (ASC 13/169 [8% seropositive] vs. SCWDS 17/169 [10% seropositive]) and <0.7 (ASC 33/169 [20% seropositive] vs. SCWDS 29/169 [17% seropositive]) (Table) (*12*). Concordance of inferred serostatus (i.e., positive/negative) of wild mammals was 95% (161/169) between laboratories when using the <0.5 S/N threshold and 89% (151/169) when using <0.7 (*12*). Twelve bELISA-negative samples were excluded from HI, VN, and ELLA assays or summary because of insufficient sample volume and sample integrity issues (*12*). When comparing HI and VN results for the remaining samples, we found comparable percentages of seropositive samples to both the HPAI H5 clade 2.3.4.4b (HI 25/157 [16%] vs. VN 36/157 [23%]) and North American lineage LPAI H5 (HI 15/157 [10%] vs. VN 26/157 [17%]) virus antigens (Table) (*12*). Inferred serostatus (positive/negative) agreed for 93% (146/157) of wild mammal serum samples tested using HI and VN for the HPAI H5 clade 2.3.4.4b virus antigen and 90% (142/157) of samples for the North American lineage LPAI H5 virus antigen (*12*). None of the serum samples collected from 33 individual mammals in Alaska before the first confirmed occurrence of HPAI H5N1 clade 2.3.4.4b in North America (November 2021) (*13*) tested positive for antibodies to H5 (HPAI or LPAI) or N1 antigens (Table) (*12*).

**Table:** Seropositivity of samples to influenza A virus antigens from exposure of wild mammals, Alaska, USA, 2020–2023*

Sample collection dates	NP bELISA+					H5 clade 2.3.4.4b+			H5 North American lineage+		N1+, ELLA§	Antibodies to H5 clade 2.3.4.4b and N1¶
	ASC, <0.5†	SCWDS <0.5†	ASC, <0.7†	SCWDS <0.7†		HI‡	VN‡		HI‡	VN‡		
2020 Jan 3–2021 Apr 5	0/34	0/34	0/34	0/34		0/33	0/33		0/33	0/33	0/33	0/33
2021 Dec 16–2023 Sep 2	13/135 (10)	17/135 (13)	33/135 (24)	29/135 (21)		25/124 (20)	36/124 (29)		15/124 (12)	26/124 (21)	34/124 (27)	33/124 (27)
All dates combined	13/169 (8)	17/169 (10)	33/169 (20)	29/169 (17)		25/157 (16)	36/157 (23)		15/157 (10)	26/157 (17)	34/157 (22)	33/157 (21)

*Values are no. positive/no. tested (%). ASC, US Geological Survey Alaska Science Center; bELISA, blocking ELISA; ELLA, enzyme-linked lectin assay; HI, hemagglutination inhibition; NP, nucleoprotein 1; SCWDS, University of Georgia Southeastern Cooperative Wildlife Disease Study; VN, virus microneutralization; +, positive. †bELISA seropositivity to the influenza A virus NP at the ASC and SCWDS determined using the manufacturer’s recommendation of serum/negative optical density of <0.5 or the threshold of <0.7 evidenced to be both sensitive and specific for wild birds (*8*,*9*). ‡HI and VN seropositivity determined by using a reverse genetic antigen (IDCDC-RG71A [H5N8]) constructed with the hemagglutinin and neuraminidase gene segments from A/Astrakhan/3212/2020 (H5N8) on PR8 backbone (clade 2.3.4.4b H5) or a reverse genetic antigen constructed with the hemagglutinin and neuraminidase gene segments from A/Blue-winged teal/AI12-4150/Texas/2012 (H5N2) on PR8 backbone (North American lineage H5). §ELLA seropositivity determined by using the antigen A/ruddy turnstone/New Jersey/AI13-2948/2013(H10N1) (N1). ¶Serum samples from mammals inhabiting Alaska testing positive for antibodies to H5 and N1 as determined by VN and ELLA.

**Figure 2:**
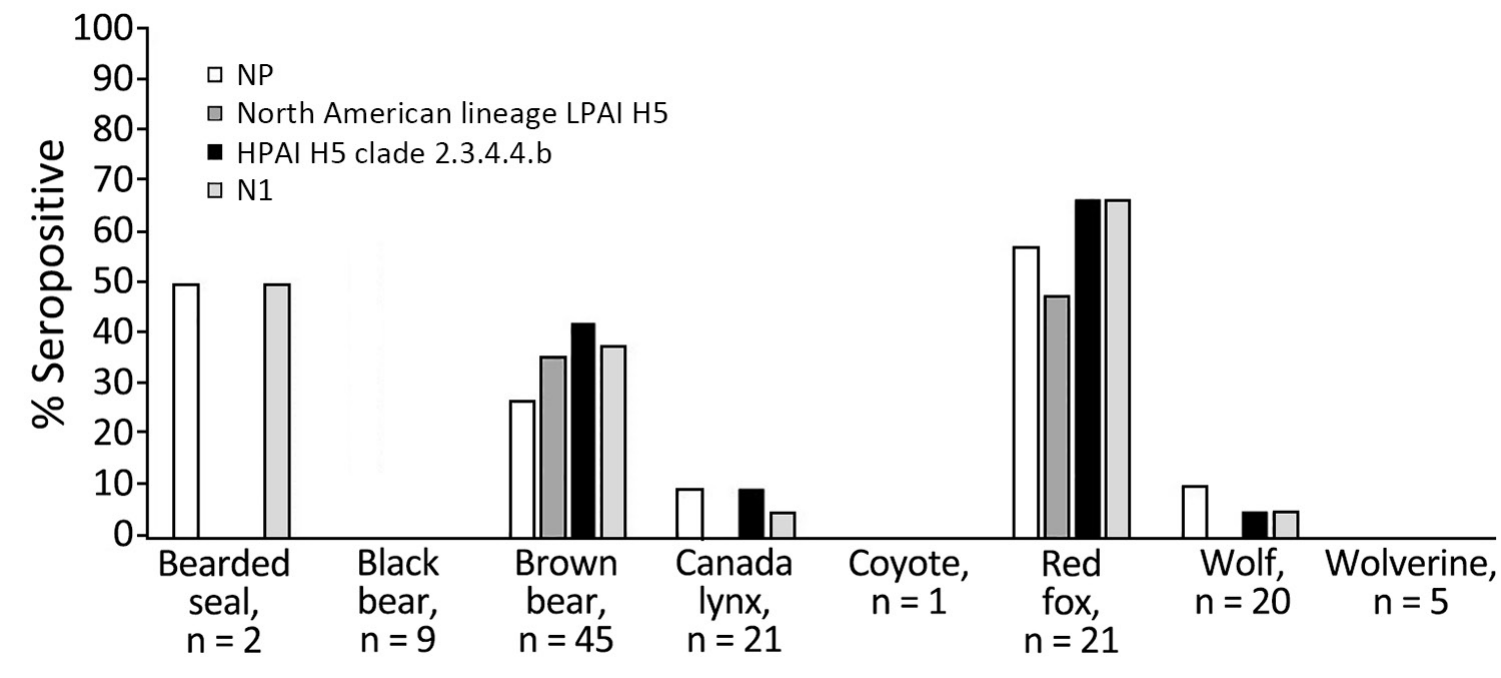
Inferred seropositivity among 124 samples collected from wild mammals, Alaska, USA, December 2021–September 2023, after detection of HPAI H5 clade 2.3.4.4b in North America, to influenza A antigens. Seropositivity to NP was determined using a blocking ELISA. Seropositivity to North American lineage LPAI H5 and HPAI H5 clade 2.3.4.4b were determined using virus microneutrialization. Seropositivity to N1 was determined using an enzyme-linked lectin assay. HPAI, highly pathogenic avian influenza; LPAI, low pathogenicity avian influenza; NP, nucleoprotein.

Antibodies to H5 and N1 subtype antigens were detected among 4 species, brown bear, Canada lynx, red fox, and wolf, by using 124 samples collected from wild mammals inhabiting Alaska after detection of HPAI H5N1 clade 2.3.4.4b in North America (Figure 2) (*12*). Samples from 33 mammals were seropositive for both the HPAI H5 clade 2.3.4.4b (using VN) and N1 antigens, including 1 sample from each Canada lynx (1/21 [5%]) and wolf (1/20 [5%]) (Figure 2) (*12*). In contrast, 38% (17/45) of brown bear and 67% (14/21) of red fox serum samples were reactive to both the HPAI H5 clade 2.3.4.4b (using VN) and N1 antigens (Figure 2) (*12*). Titers of antibodies reactive to the HPAI H5 clade 2.3.4.4b antigen were higher than the LPAI H5 antigen for most of those samples using VN (28/33 [85%]) (*12*). The geometric mean titer of H5 and N1 seropositive brown bear samples for the HPAI H5 clade 2.3.4.4b antigen (using VN) was 154 and for the N1 antigen was 694 (*12*). The geometric mean titer of seropositive red fox samples was 1,159 for the HPAI H5 clade 2.3.4.4b antigen and 706 for the N1 antigen (*12*). Brown bears inferred to be seropositive for HPAI H5 clade 2.3.4.4b (using VN) and N1 antigens were sampled along the Arctic North Slope of Alaska (n = 15) and Lake Clark National Park and Preserve (n = 2), whereas all HPAI H5 clade 2.3.4.4b and N1 seropositive red foxes were sampled in the Northwest Arctic (Figure 1) (*12*).

## Conclusions

Serologic data from diverse opportunistically sampled wild mammals inhabiting Alaska provide insights into the exposure of nonreservoir wildlife species to HPAI H5N1 clade 2.3.4.4b viruses, immune responses, and possible outcomes of infection. For example, for some wild mammal species, such as brown bears and red foxes, a relatively large proportion of animals in Alaska might have been exposed to H5N1 viruses within specific regions and contexts. Most H5 and N1 seropositive bear and fox samples originated from regions with extensive wetland complexes and where wild aquatic birds had been confirmed to be infected with HPAI H5N1 clade 2.3.4.4b viruses (*14*). Those serologic data also provide evidence that HPAI H5N1 clade 2.3.4.4b infections may not always result in fatal outcomes among wild mammals; some animals apparently mount sufficient immune responses to overcome infection. Verification that H5 and N1 antibodies were acquired from infections with HPAI H5N1 clade 2.3.4.4b viruses is not possible because cross-reactivity with other antigens might have occurred. Nonetheless, the combination of high titers to the HPAI H5 clade 2.3.4.4b and N1 antigens among seropositive samples (suggestive of close antigenic match), comparably lower titers to the North American LPAI H5 antigen (suggestive of more distant antigenic match), and spatiotemporal context of seropositive samples (*12*) supports probable exposure to HPAI H5N1 clade 2.3.4.4b viruses.

Additional research is needed to identify factors affecting individual and species-specific susceptibility to infection, manifestation of clinical disease, role of preexisting immunity, and duration of detectable immune response among wild mammals. In the absence of more comprehensive assessments of exposure of wild mammals to HPAI H5N1 clade 2.3.4.4b viruses, caution might be prudent in any extrapolation of information we present to other geographic areas or other species without careful consideration of epidemiologic context. Future surveillance efforts may be improved by incorporating information on susceptibility and detectable immune responses among wild mammals.
